# Development of a Machine Learning-Based Predictive Model for Lung Metastasis in Patients With Ewing Sarcoma

**DOI:** 10.3389/fmed.2022.807382

**Published:** 2022-04-01

**Authors:** Wenle Li, Tao Hong, Wencai Liu, Shengtao Dong, Haosheng Wang, Zhi-Ri Tang, Wanying Li, Bing Wang, Zhaohui Hu, Qiang Liu, Yong Qin, Chengliang Yin

**Affiliations:** ^1^Department of Orthopedics, Xianyang Central Hospital, Xianyang, China; ^2^Clinical Medical Research Center, Xianyang Central Hospital, Xianyang, China; ^3^Department of Cardiac Surgery, Fuwai Hospital Chinese Academy of Medical Sciences, Shenzhen, Shenzhen, China; ^4^Department of Orthopaedic Surgery, the First Affiliated Hospital of Nanchang University, Nanchang, China; ^5^Department of Spine Surgery, Second Affiliated Hospital of Dalian Medical University, Dalian, China; ^6^Department of Orthopaedics, The Second Hospital of Jilin University, Changchun, China; ^7^School of Physics and Technology, Wuhan University, Wuhan, China; ^8^Department of Spinal Surgery, Liuzhou People's Hospital, Liuzhou, China; ^9^Department of Orthopedics Surgery, The Second Affiliated Hospital of Harbin Medical University, Harbin, China; ^10^Faculty of Medicine, Macau University of Science and Technology, Macau, Macau SAR, China

**Keywords:** Ewing sarcoma, lung metastasis, machine learning algorithms, multicenter, web calculator

## Abstract

**Background:**

This study aimed to develop and validate machine learning (ML)-based prediction models for lung metastasis (LM) in patients with Ewing sarcoma (ES), and to deploy the best model as an open access web tool.

**Methods:**

We retrospectively analyzed data from the Surveillance Epidemiology and End Results (SEER) Database from 2010 to 2016 and from four medical institutions to develop and validate predictive models for LM in patients with ES. Patient data from the SEER database was used as the training group (*n* = 929). Using demographic and clinicopathologic variables six ML-based models for predicting LM were developed, and internally validated using 10-fold cross validation. All ML-based models were subsequently externally validated using multiple data from four medical institutions (the validation group, *n* = 51). The predictive power of the models was evaluated by the area under receiver operating characteristic curve (AUC). The best-performing model was used to produce an online tool for use by clinicians to identify ES patients at risk from lung metastasis, to improve decision making and optimize individual treatment.

**Results:**

The study cohort consisted of 929 patients from the SEER database and 51 patients from multiple medical centers, a total of 980 ES patients. Of these, 175 (18.8%) had lung metastasis. Multivariate logistic regression analysis was performed with survival time, T-stage, N-stage, surgery, and bone metastasis providing the independent predictive factors of LM. The AUC value of six predictive models ranged from 0.585 to 0.705. The Random Forest (RF) model (*AUC* = 0.705) using 4 variables was identified as the best predictive model of LM in ES patients and was employed to construct an online tool to assist clinicians in optimizing patient treatment. (https://share.streamlit.io/liuwencai123/es_lm/main/es_lm.py).

**Conclusions:**

Machine learning were found to have utility for predicting LM in patients with Ewing sarcoma, and the RF model gave the best performance. The accessibility of the predictive model as a web-based tool offers clear opportunities for improving the personalized treatment of patients with ES.

## Introduction

Ewing sarcoma (ES) is an aggressive sarcoma with a high propensity for local recurrence and distant metastasis in children and adolescents (1, 2). ES is the second most common primary bone malignancy, accounting for 5% of all child and adolescent cancers (3). ES frequently involves the diaphysis region of long bones (4). Despite the development of new treatment regimens, ES has a high likelihood of tumor metastasis, leading to a worsening prognosis and resulting in a poor 5-year survival rate of only 20–45% (4, 5). In a retrospective study of 975 patients with ES, 5-year survival and 5-year relapse-free survival rates for patients with localized disease were 70 and 55%, respectively, but only 33 and 21% for those with distant metastasis disease (6).

Although diagnostic imaging techniques have improved dramatically during the past 30 years, metastatic status can only be detected in approximately 20–25% of ES patients (3), with the lung being the most common metastatic site (5, 7, 8). Computed tomography (CT) scans of the chest are usually carried out to detect lung metastasis. However, given the high cost, radiation damage, and low efficiency of detection of metastatic nodules, new strategies are urgently required to accurately predict the development of lung metastasis in patients with ES (9, 10).

Machine learning (ML) has emerged as a powerful computer-based method of data mining and analysis and has been extensively applied as a “prediction tool” in a multitude of different scientific, engineering, and medical scenarios (11–15). ML has been shown to detect more interactions between variables, and to be more accurate than conventional statistical methods (14, 16). ML algorithms have been applied to model clinical outcome and to improve cognition of tumor growth and progression (17). However, although numerous ML-based predictive models of tumor development have been reported, no study has been conducted in predicting lung metastasis associated with Ewing Sarcoma.

The Surveillance Epidemiology and End Results (SEER) database contains data for around 26% of the United States population and is commonly used to study rare diseases since it overcomes the obstacle of inadequate case numbers (18–20). We constructed several ML-based models of LM in patients with ES, using the SEER database. External validation was subsequently performed using data from multiple medical centers to predict the probability of LM with the aim of improving individualized patient management. The best model was uploaded as a web-based tool.

## Materials and Methods

### Study Population and Data Selection

Data were sourced from the SEER database and four medical institutions in China: Liuzhou People's Hospital, Second Affiliated Hospital of Jilin University, Xianyang Central Hospital, and Second Affiliated Hospital of Dalian Medical University, respectively. This retrospective study did not use personal identifying information and thus did not require informed patient consent or Institutional Ethics Committee Board approval.

Patients selected from the SEER database (2010–2016) who were diagnosed with ES originating in bone, as identified by ICD-O-3/WHO 2008 morphology code 9260d, composed the “training” group. Criteria for exclusion were more than one primary tumor and incomplete clinicopathological information. The “validation” group was composed of ES patient data obtained from four hospitals in different regions of China, from 2010 to 2018. All cases featured complete clinicopathological data and follow-up information and no other primary tumors. Demographic and clinicopathological variables included in both groups were: race, age, sex, primary site, laterality, T-stage, N-stage, M-stage, surgery, radiation, chemotherapy, bone metastasis, and survival times. For consistency with SEER database records, “race” in the Chinese medical records was classified as “other”. Detailed treatments, such as surgery, radiation, and chemotherapy were classified as Yes or No, and were not recorded in the SEER database.

### Establishment and Evaluation of Prediction Models

Using demographic and clinicopathological data, we explored the effect of variables (*p* < 0.05) in univariate analysis, in the multifactorial regression model, and in predictive models based on the ML algorithms. Six different ML algorithms were applied independently to develop predictive models of LM in patients with ES, as follows: Random Forest (RF), Logistic regression (LR), Extreme gradient boosting (XGB), Gradient boosting machine (GBM), Multilayer perceptron (MLP), and Decision tree (DT) (21, 22). For the training process of the ML algorithms using python (version 3.8), we employed 10-fold cross-validation to avoid overfitting (23). We also calculated the average value of the area under receiver operating characteristic curve (AUC) to evaluate the predictive power of each model.

The ML algorithms were subsequently applied to the validation group and the AUC was again calculated to evaluate the predictive performance of all models. The higher the AUC value, the better the model. Finally, the best-performing model was designed as a web-based tool for predicting the likelihood of LM in ES patients.

As a model inspection technique, permutation feature importance can be used for any fitted estimator (24–26). Thus, a total of 100 independent training simulation results were applied to assess the most important variables in each predictive model using permutation feature importance analysis. We further assessed the relative contribution of four key clinical variables to LM predictive models using spearman correlation of features analysis and plotted a correlation heat map.

### Statistical Analysis

All data were extracted from the SEER database *via* the SEER ^*^ Stat software (version 8.3.6). All analyses were performed using python (version 3.8). The baseline variables between the training group and validation group were compared using Student's *t* tests and Pearson chi-square test. A two-sided *p* < 0.05 was deemed to have statistical significance.

## Results

### Baseline Characteristics

A total of 980 patients with ES were enrolled in this study; 929 patients originating from the SEER database were assigned to the training group; and 51 patients from four medical centers in China were assigned to the validation group (Table 1). There were significant differences between the two groups in terms of race, T-stage, and radiation (*p* < 0.05). In the validation group, all patients were classified under race as “others”. The proportion of radiation was significantly higher in the validation group than in the training group. In addition, more patients were diagnosed as TX in the training group. The remaining variables were not significantly different in both groups (Table 1). Lung metastasis occurred in 185 (18.9%) cases, the median age of the patients was 22.25 years (*SD* = 16.3), more than 85% of the patients were Caucasian and 534 (57.5%) patients were male. Comparison of the baseline data between the lung metastasis group and no lung metastasis group, revealed significant differences for the following factors: T-stage, N-stage, M-stage, surgery, bone metastasis, and survival time (*p* < 0.001). The demographic and clinicopathological variables of all 980 patients are summarized in Table 2.

**Table 1:** Baseline of patients with SEER database and multicenter data.

	**level**	**Overall (*N* = 980)**	**Multicenter (validation group, *N* = 51)**	**SEER (training group, *N* = 929)**	**p**
Race (%)	Black	39 (4.0)	0 (0.0)	39 (4.2)	<0.001
	Other	126 (12.9)	51 (100.0)	75 (8.1)	
	White	815 (83.2)	0 (0.0)	815 (87.7)	
Age [mean (SD)]	NA	22.39 (16.45)	24.96 (18.97)	22.25 (16.30)	0.252
Sex (%)	Female	418 (42.7)	23 (45.1)	395 (42.5)	0.828
	Male	562 (57.3)	28 (54.9)	534 (57.5)	
Primary. Site (%)	Axis bone	431 (44.0)	27 (52.9)	404 (43.5)	0.394
	Limb bone	317 (32.3)	13 (25.5)	304 (32.7)	
	other	232 (23.7)	11 (21.6)	221 (23.8)	
Laterality (%)	left	374 (38.2)	21 (41.2)	353 (38.0)	0.894
	Not a paired site	296 (30.2)	15 (29.4)	281 (30.2)	
	right	310 (31.6)	15 (29.4)	295 (31.8)	
T (%)	T1	351 (35.8)	20 (39.2)	331 (35.6)	0.008
	T2	429 (43.8)	25 (49.0)	404 (43.5)	
	T3	39 (4.0)	5 (9.8)	34 (3.7)	
	TX	161 (16.4)	1 (2.0)	160 (17.2)	
N (%)	N0	841 (85.8)	44 (86.3)	797 (85.8)	0.312
	N1	80 (8.2)	6 (11.8)	74 (8.0)	
	NX	59 (6.0)	1 (2.0)	58 (6.2)	
M (%)	M0	662 (67.6)	30 (58.8)	632 (68.0)	0.225
	M1	318 (32.4)	21 (41.2)	297 (32.0)	
surgery (%)	No	413 (42.1)	25 (49.0)	388 (41.8)	0.381
	Yes	567 (57.9)	26 (51.0)	541 (58.2)	
Radiation (%)	No	757 (77.2)	29 (56.9)	728 (78.4)	0.001
	Yes	223 (22.8)	22 (43.1)	201 (21.6)	
Chemotherapy (%)	No/Unknown	58 (5.9)	0 (0.0)	58 (6.2)	0.125
	Yes	922 (94.1)	51 (100.0)	871 (93.8)	
Bone.metastases (%)	No	831 (84.8)	40 (78.4)	791 (85.1)	0.271
	Yes	149 (15.2)	11 (21.6)	138 (14.9)	
Lung.metastases (%)	No	795 (81.1)	41 (80.4)	754 (81.2)	1
	Yes	185 (18.9)	10 (19.6)	175 (18.8)	
times [mean (SD)]	NA	30.56 (22.65)	29.71 (22.40)	30.61 (22.67)	0.782

**Table 2:** Baseline table of patients in the Ewing sarcoma lung metastasis group vs. the no lung metastasis group.

	**Level**	**Overall (*N* = 929)**	**No (*N* = 754)**	**Yes (*N* = 175)**	***p***
Race (%)	Black	39 (4.2)	27 (3.6)	12 (6.9)	0.105
	Other	75 (8.1)	64 (8.5)	11 (6.3)	
	White	815 (87.7)	663 (87.9)	152 (86.9)	
Age [mean (SD)]	NA	22.25 (16.30)	22.10 (16.35)	22.88 (16.10)	0.569
Sex (%)	Female	395 (42.5)	329 (43.6)	66 (37.7)	0.18
	Male	534 (57.5)	425 (56.4)	109 (62.3)	
Primary.Site (%)	Axis bone	404 (43.5)	316 (41.9)	88 (50.3)	0.13
	Limb bone	304 (32.7)	253 (33.6)	51 (29.1)	
	other	221 (23.8)	185 (24.5)	36 (20.6)	
Race (%)	Black	39 (4.2)	27 (3.6)	12 (6.9)	0.105
	Other	75 (8.1)	64 (8.5)	11 (6.3)	
	White	815 (87.7)	663 (87.9)	152 (86.9)	
T (%)	T1	331 (35.6)	304 (40.3)	27 (15.4)	<0.001
	T2	404 (43.5)	312 (41.4)	92 (52.6)	
	T3	34 (3.7)	20 (2.7)	14 (8.0)	
	TX	160 (17.2)	118 (15.6)	42 (24.0)	
N (%)	N0	797 (85.8)	676 (89.7)	121 (69.1)	<0.001
	N1	74 (8.0)	37 (4.9)	37 (21.1)	
	NX	58 (6.2)	41 (5.4)	17 (9.7)	
M (%)	M0	632 (68.0)	632 (83.8)	0 (0.0)	<0.001
	M1	297 (32.0)	122 (16.2)	175 (100.0)	
surgery (%)	No	388 (41.8)	271 (35.9)	117 (66.9)	<0.001
	Yes	541 (58.2)	483 (64.1)	58 (33.1)	
Radiation (%)	No	728 (78.4)	593 (78.6)	135 (77.1)	0.739
	Yes	201 (21.6)	161 (21.4)	40 (22.9)	
Chemotherapy (%)	No/Unknown	58 (6.2)	45 (6.0)	13 (7.4)	0.585
	Yes	871 (93.8)	709 (94.0)	162 (92.6)	
Bone.metastases (%)	No	791 (85.1)	672 (89.1)	119 (68.0)	<0.001
	Yes	138 (14.9)	82 (10.9)	56 (32.0)	
times [mean (SD)]	NA	30.61 (22.67)	32.40 (22.83)	22.89 (20.31)	<0.001

### Univariate and Multifactorial LR Analysis of LM

The following variables were shown to have significant correlation with the development of LM in univariate analysis (*p* < 0.05): survival time, T-stage, N-stage, surgery, and bone metastasis (*p* < 0.001) (Table 3). Multifactorial LR analysis based on the variables (*p* < 0.05) in univariate analysis, demonstrated that T- stage (T2, *OR* = 2.7018, 95% *CI* = 1.690–4.317; T3, *OR* = 4.0378, 95% *CI* = 1.773–9.194; TX, *OR* = 3.1468, 95% *CI* = 1.778–5.566), N1 stage [*vs*. N0 stage, N1, (*OR* = 5.102, 95% *CI* = 3.048–8.540)], and bone metastasis (*OR* = 1.685, 95% *CI* = 1.090–2.605) were independent negative predictors of LM while survival time (*OR* = 0.988, 95% *CI* = 0.979–0.997) and surgery (*OR* = 0.451, 95% *CI* = 0.309–0.658) were positive predictors.

**Table 3:** Univariate and multifactorial logistic regression analysis of risk factors for lung metastasis in patients with Ewing sarcoma.

**Variables**	**Univariate OR (95% CI)**	***p* value**	**Multivariate OR (95% CI)**	***p* value**
Age (years)	1.000 (0.991–1.010)	0.968	/	/
Survival time (month)	0.980 (0.973–0.988)	<0.001	0.988 (0.979–0.997)	<0.01
**Race**				
White	Ref	Ref	Ref	Ref
Black	1.939 (0.960–3.914)	0.065	/	/
Other	0.872 (0.529–1.439)	0.593	/	/
**Sex**				
Male	Ref	Ref	Ref	Ref
Female	0.804 (0.579–1.116)	0.192	/	/
**Primary site**				
Limb bones	Ref	Ref	Ref	Ref
Axis of a bone	1.359 (0.937–1.970)	0.106	/	/
other	0.924 (0.585–1.460)	0.735	/	/
**Laterality**				
Left	Ref	Ref	Ref	Ref
Right	1.148 (0.784–1.681)	0.479	/	/
Other	1.004 (0.676–1.491)	0.984	/	/
**T**				
T1	Ref	Ref	Ref	Ref
T2	3.461 (2.214–5.410)	<0.001	2.701 (1.690–4.317)	<0.001
T3	8.025 (3.8074–16.917)	<0.001	4.037 (1.773–9.194)	<0.01
TX	4.071 (2.415–6.864)	<0.001	3.146 (1.778–5.566)	<0.001
**N**				
N0	Ref	Ref	Ref	Ref
N1	5.570 (0.3457–8.975)	<0.001	5.102 (3.048–8.540)	<0.001
NX	2.255 (1.245–4.084)	<0.01	1.411 (0.734–2.715)	0.302
**Surgery**				
No	Ref	Ref	Ref	Ref
Yes	0.278 (0.196–0.394)	<0.001	0.451 (0.309–0.658)	<0.001
**Radiation**				
No	Ref	Ref	Ref	Ref
Yes	1.241 (0.858–1.795)	0.251	/	/
**Chemotherapy**				
No	Ref	Ref	Ref	Ref
Yes	0.794 (0.419–1.504)	0.479	/	/
**Bone metastases**				
No	Ref	Ref	Ref	Ref
Yes	3.403 (2.326–4.977)	<0.001	1.685 (1.090–2.605)	<0.05

### Predictive Performance of Machine Learning (ML) Algorithms

**Figure 1:**
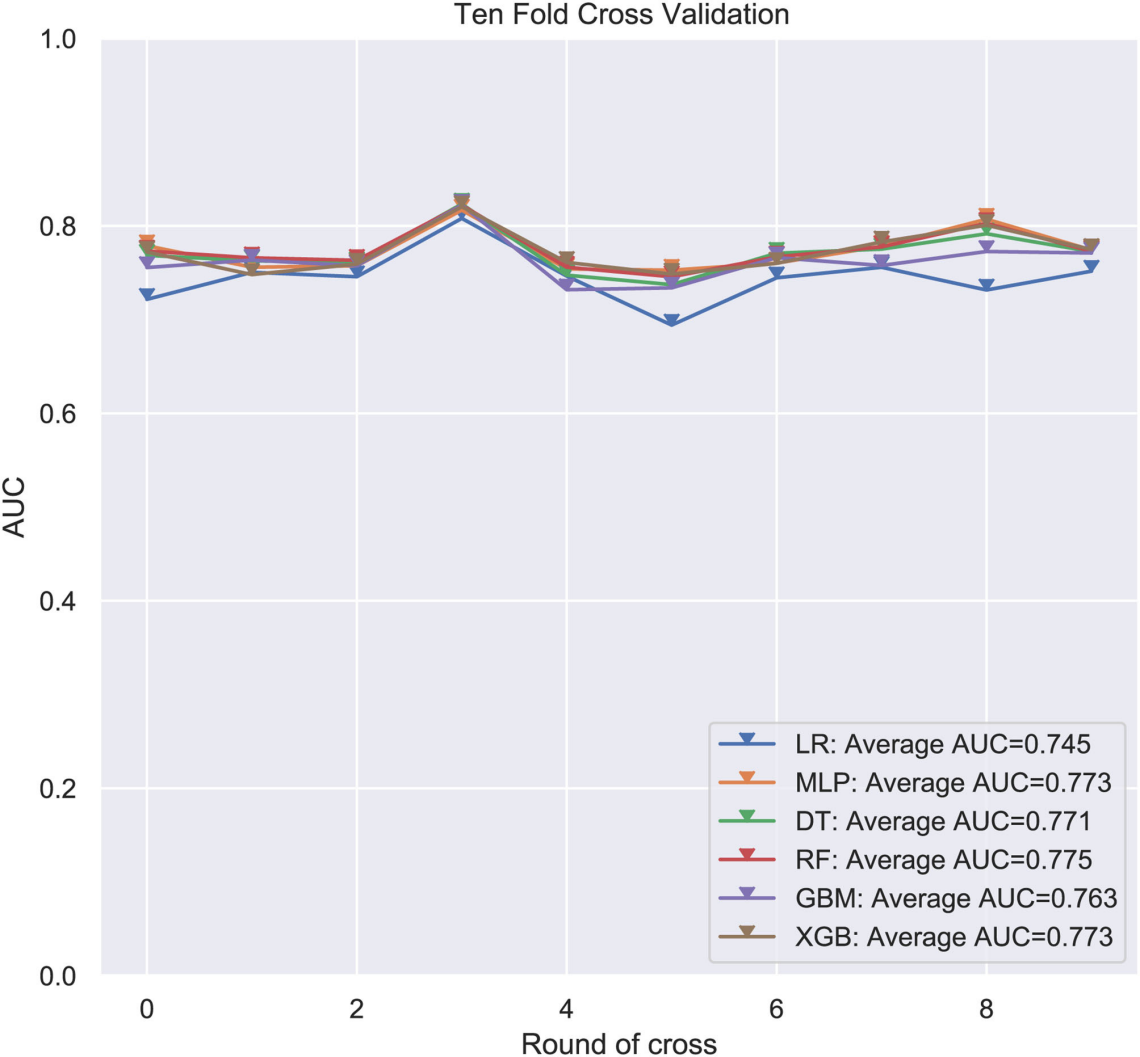
Average area under the curve (AUC) values of 10-fold cross-validation. RF, Random forest predictive model; DT, Decision tree; XGB, Extreme gradient boosting; GBM, Gradient boosting machine; MLP, Multilayer perceptron; LR, Logistic regression; AUC used as an indicator of performance, RF model achieved the best predictive performance while the MLP model showed the lowest.

**Figure 2:**
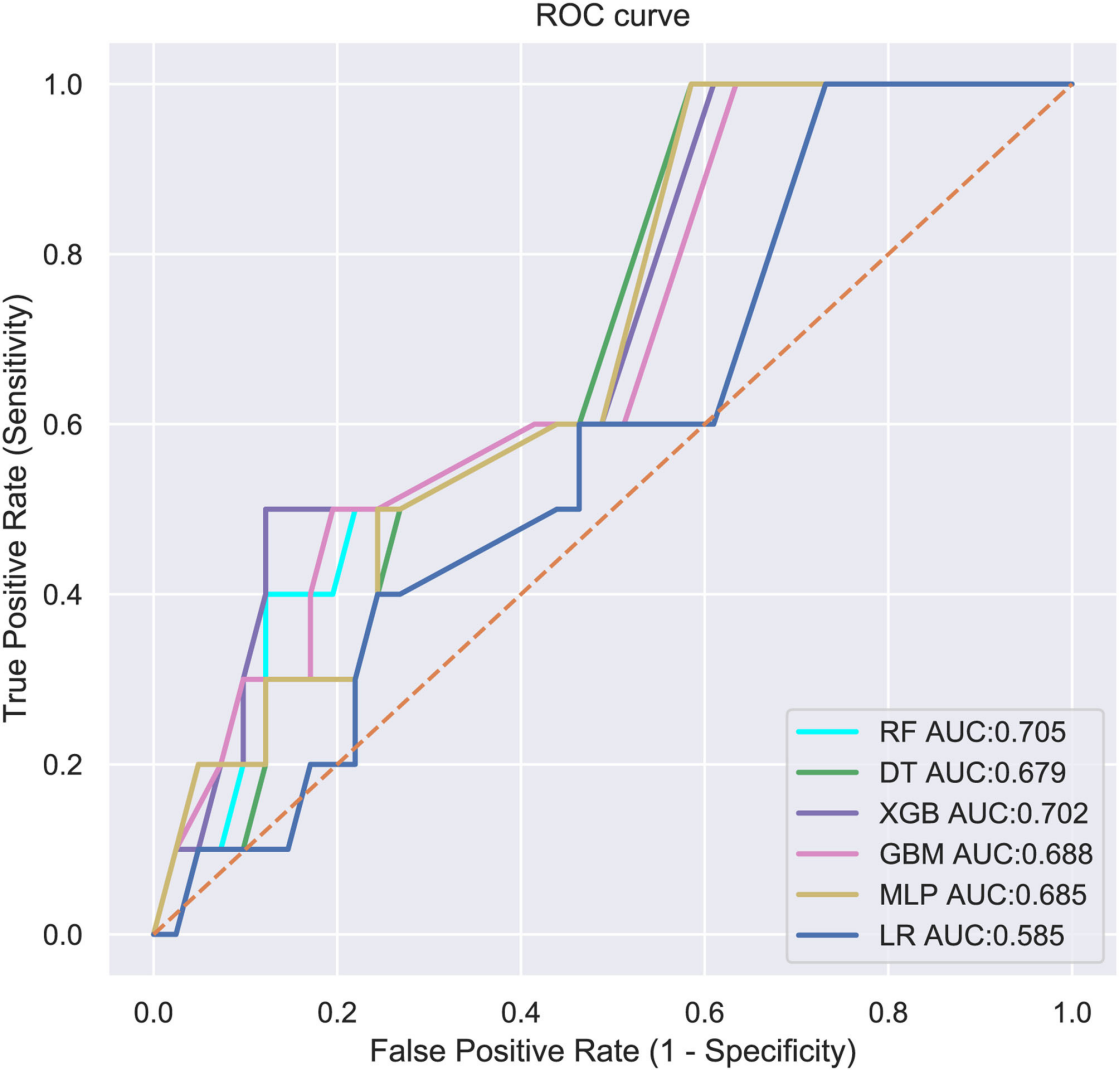
External validation of machine learning algorithms. RF, Random Forest; DT, Decision tree; XGB, Extreme gradient boosting; GBM, Gradient boosting machine; MLP, Multilayer perceptron; LR, Logistic regression; AUC, area under the curve.

Six ML-based models for predicting LM in ES patients were developed based on the training group data. The average AUC of the six models determined by 10-fold cross-validation is shown in Figure 1, with the RF model achieving the best performance (*AUC* = 0.775). When the models established in training were subjected to external validation (Figure 2), the RF model still achieved the best performance (*AUC* = 0.705) in predicting LM and was accordingly selected as the design for a web-based, predictive tool.

### Influence of Variables on Prediction Performance

**Figure 3:**
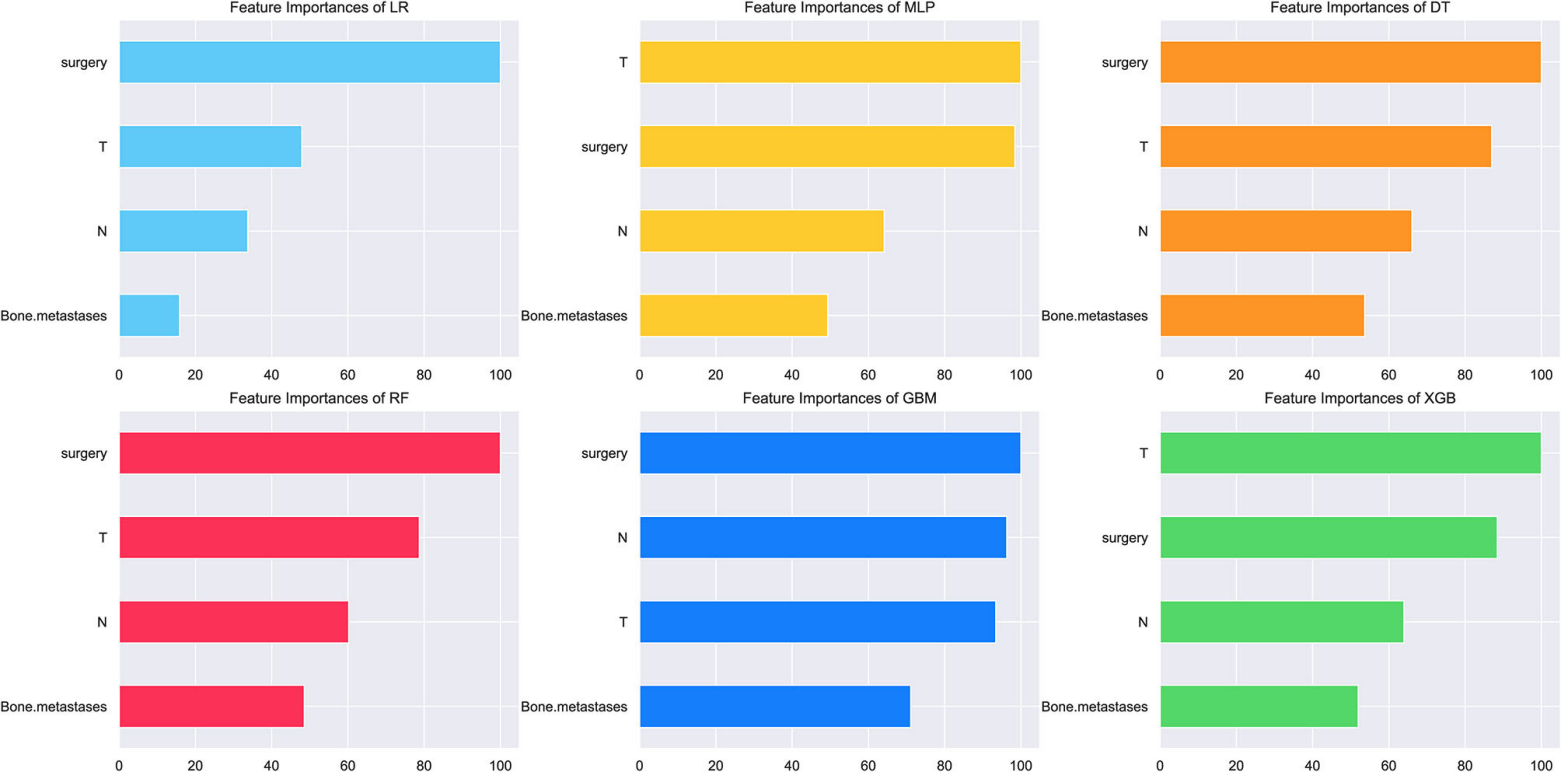
The relative importance of variables for the prediction of LM using ML algorithms. Surgery, T-stage and N-stage ranked in the top three in all prediction models, with bone metastasis ranked fourth.

**Figure 4:**
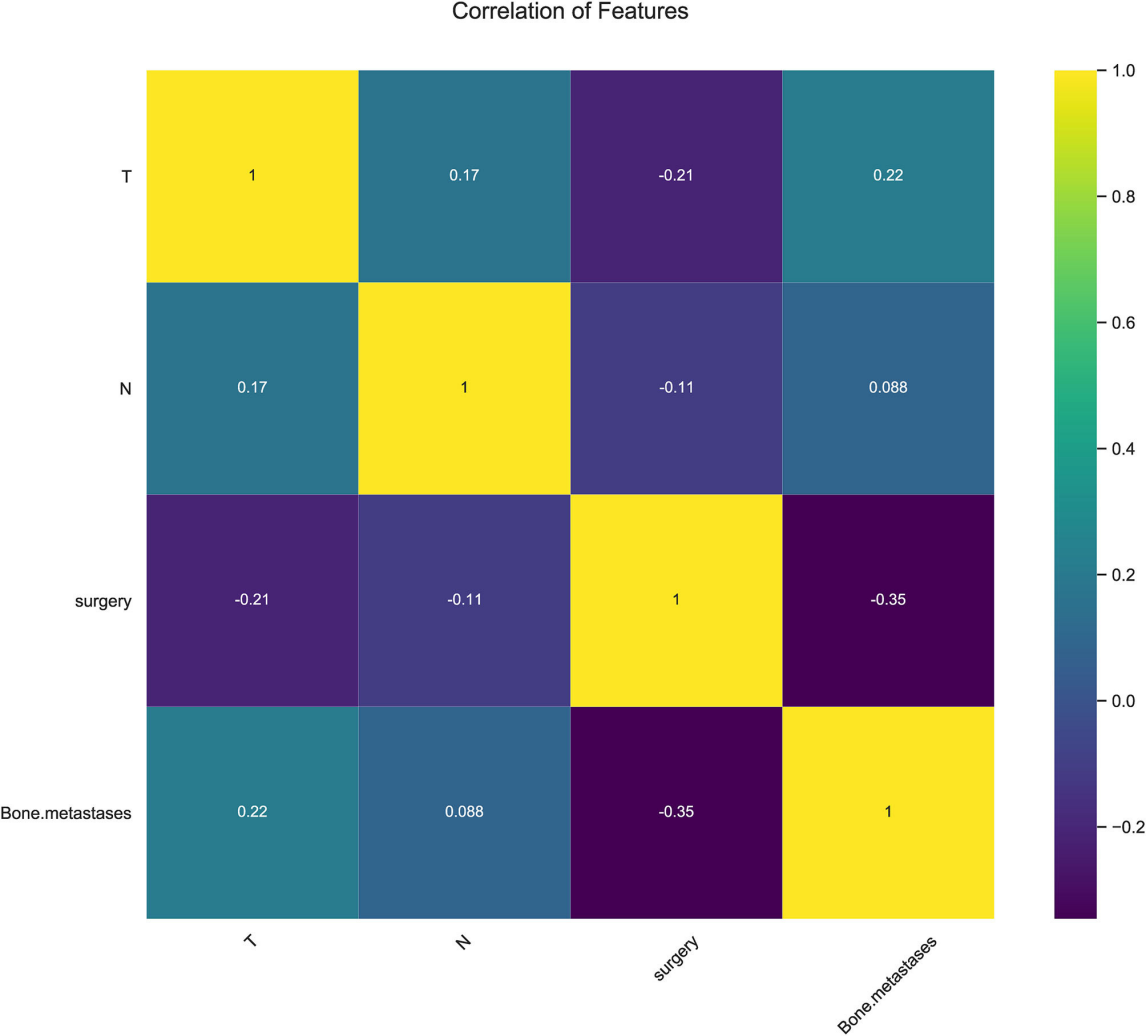
Results of Pearson correlation of features analysis between all variables showing no obvious correlation between every two variables.

In consideration of clinical utility (Figure 3), we focused on four variables (T-stage, N-stage, surgery, and bone metastasis) to construct ML-based predictive models for LM in ES patients. Although there were slight differences in the importance of variables identified by each model; three factors, such as surgery, T-stage and N-stage, consistently ranked in the top three, and bone metastasis ranked fourth. The relative importance of variables in predicting LM using the RF model decreased in the order: surgery > T-stage > N-stage > bone metastasis. Analysis using spearman correlation of features approach revealed no significant positive correlation between any variable, and a negative correlation between surgery and the other three variables, indicating that all variables were independent (Figure 4).

### Design of a Web-Based Tool for Predicting LM in ES Patients

**Figure 5:**
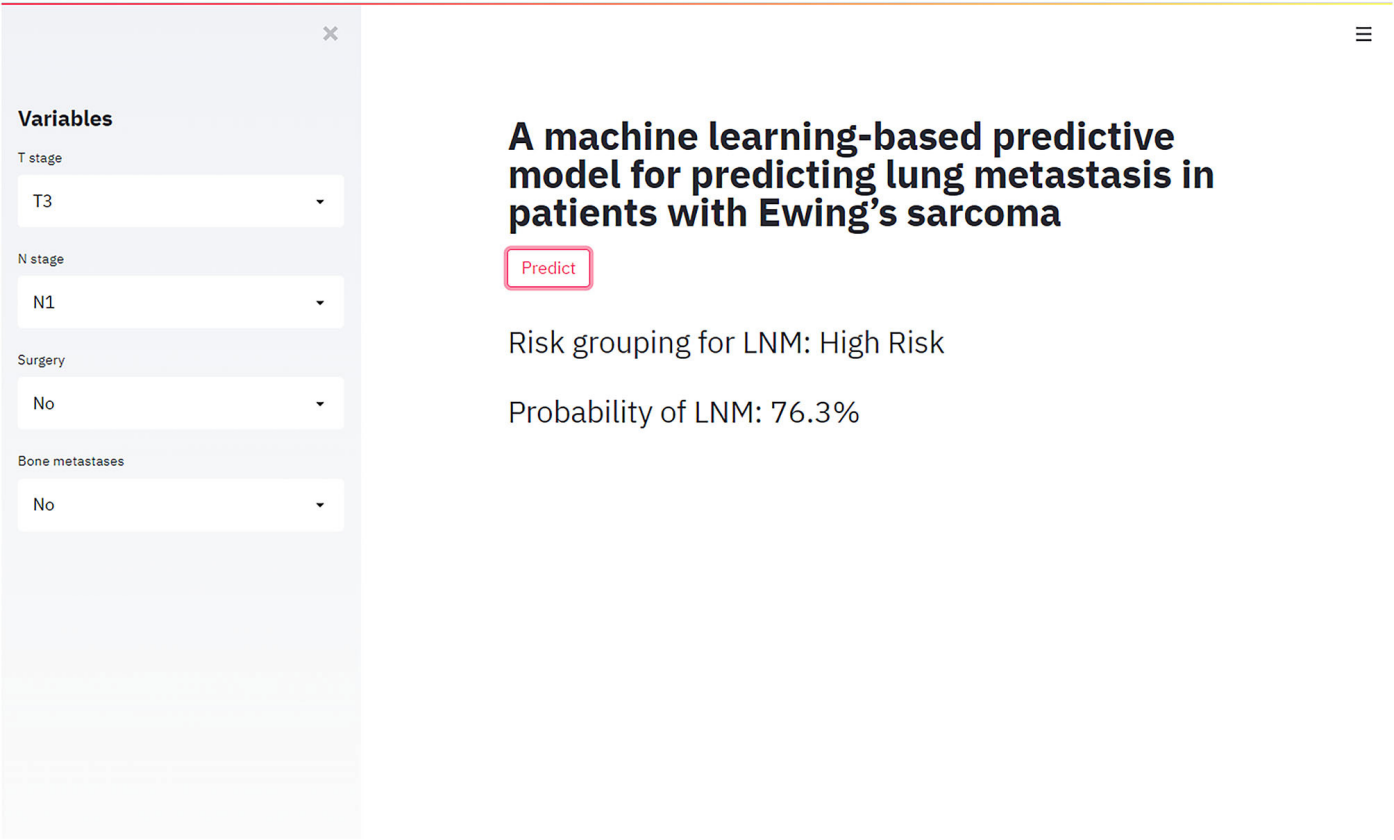
The web-based tool designed for predicting lung metastasis in patients with Ewing sarcoma.

The best-performing RF model was used to design a web-based tool to assist clinicians in predicting lung metastasis in ES patients (https://share.streamlit.io/liuwencai123/es_lm/main/es_lm.py) (Figure 5).

## Discussion

Multi-modal therapy of metastatic disease based on chemotherapy, surgery, and radiation would be improved dramatically by the availability of reliable methods for predicting metastasis (27, 28). Many mathematical models of tumor malignancy employ multivariate regression or correlation analysis, which usually require the variables to be independent and linear (29–32). In addition to traditional univariate and multivariate analysis, we used multiple ML algorithms, which are widely applied in healthcare data analysis, to construct predictive models of LM in ES patients. We found that the RF model provided the best performance. RF is a commonly used ML algorithm that has a proven track record in handling large complex nonlinear datasets (33, 34). We subsequently designed a rapid web-based clinical tool, which is based on the RF model, for predicting lung metastasis in patients with ES.

Patient survival time was positively related to LM in univariate analysis. However, when considering clinical practice, survival time has no meaning for patients initially diagnosed with ES, and it is difficult to assess the survival time of a part of the patient population. Thus, survival time was not considered as a variable in ML models.

In the present study, four clinical variables: surgery, T-stage, N-stage, and bone metastasis were found to be the most important factors for predicting LM status by ML algorithms. We identified surgery as a protective factor against LM. To our knowledge, this factor has not been included previously in LM risk prediction models. Surgery is not only a vital form of treatment, but also plays a significant diagnostic role, which enables more accurate TNM staging and prognosis of ES patients. Surgery ranked first in order of importance in most of the predictive models developed in the present study, while T-stage (tumor size) ranked in the top two in all models investigated and was highly predictive of LM, similar to previous reports (35, 36). Large tumor volume indicates a longer growth cycle, resulting in a more proliferative and aggressive state, thus increasing the occurrence of lung metastasis. The correlation heat map showed that the T-stage correlated negatively with surgery since radical surgical treatment is difficult for large tumors, and lung metastasis is more likely.

Extensive investigations have consistently demonstrated that patients with regional node involvement were more prone to develop distant metastasis (37–41). Since the lung is associated with an abundance of lymphatic vessels, a tumor is more likely to metastasize to the lung when lymph nodes are positive. However, due to the scarcity of lymphatic vessels in bone tumor, it is conventionally accepted that dissemination to lymph nodes is uncommon (4, 42). Applebaum et al., for example, found that only 6.3% (91/1,452) of cases featured lymph node involvement (37). In contrast, our study revealed a much higher rate of lymph node metastasis, approximately 18.9% (185/980).

Importantly, our ML-based models revealed that bone metastasis was an important predictor of LM in ES patients, ranking fourth in importance behind surgery, T-stage and N-stage variables. Of the 138 patients in the two combined cohorts (training group and validation group) who had bone metastasis, 40.6% (56/138) also displayed lung metastasis. This figure was significantly higher than the number of patients who showed LM without bone metastasis (15%, 119/791).

Our present study of ML-based models for predicting LM in ES patients contained certain limitations which, nonetheless, serve as a guide for future improvements. Firstly, the information accessed from the SEER database was to a certain degree limited. Clinical information, such as the precise surgical treatment, surgical margin status, tumor marker, vascular invasion, radiation dosage, and chemotherapy modalities were unavailable, which limits the predictive value of the developed models. Secondly, the data from the SEER database was retrospective, which may introduce bias in data selection. However, while cognizant of these limitations, our study affirmed that ML-based prediction models can effectively identify the likelihood of LM in patients with ES by inspection of clinical factors such as surgery, N-stage, T-stage, and bone metastasis. The RF model performed best according to ROC analysis and was subsequently used to produce a web-based tool designed to help clinicians identify ES patients with lung metastasis, improve decision making and optimize individual treatment. Increased case data and multicenter studies are anticipated to lead to improvements in predictive performance.

## Conclusion

Machine learning algorithms were applied to develop a prognostic tool for predicting the risk of LM in patients with ES. A RF model performed best and was engineered as a web-based tool for use by clinicians to improve patient diagnosis and treatment.

## Data Availability Statement

The raw data supporting the conclusions of this article will be made available by the authors, without undue reservation.

## Author Contributions

CY, QL, and YQ designed the study. WL and TH collected and evaluated the data and wrote the first draft of the manuscript. All authors contributed to the interpretation of the results and the final draft of the manuscript.

## Funding

This study was supported by the National Clinical Research Center for Orthopedics, Sports Medicine and Rehabilitation, and the Jiangsu China-Israel Industrial Technical Research Institute Foundation, 2021-NCRC-CXJJ-ZH-11.

## Conflict of Interest

The authors declare that the research was conducted in the absence of any commercial or financial relationships that could be construed as a potential conflict of interest.

## Publisher's Note

All claims expressed in this article are solely those of the authors and do not necessarily represent those of their affiliated organizations, or those of the publisher, the editors and the reviewers. Any product that may be evaluated in this article, or claim that may be made by its manufacturer, is not guaranteed or endorsed by the publisher.
